# Peroxisome Proliferator-Activated Receptors and Hepatitis C Virus-Induced Insulin Resistance

**DOI:** 10.1155/2009/483485

**Published:** 2009-01-06

**Authors:** Francesco Negro

**Affiliations:** Viropathology Unit, University of Geneva Medical Center, 1211 Geneva, Switzerland

## Abstract

Insulin resistance and type 2 diabetes are associated with hepatitis C virus infection. A wealth of clinical and experimental data suggests that the virus is directly interfering with the insulin signalling in hepatocytes. In the case of at least one viral genotype (the type 3a), insulin resistance seems to be directly mediated by the downregulation of the peroxisome proliferator-activated receptor *γ*. Whether and how this interaction may be manipulated pharmacologically, in order to improve the responsiveness to antivirals of insulin resistant chronic hepatitis C, patients remain to be fully explored.

## 1. Introduction

Hepatitis C virus (HCV) infection is associated with an increased risk of developing glucose intolerance and diabetes. This is in part due to a direct interference of HCV with the insulin signalling pathway. The mechanisms involved seem to be HCV genotype-specific, and this suggests that HCV may profit from the insulin resistant phenotype to establish and/or maintain a persistent infection. Since the peroxisome proliferator-activated receptors (PPARs) are nuclear factors involved—among others—in the regulation of glucose homeostasis, the relationship between HCV replication and protein expression and PPARs has been the focus of some recent studies. However, the data available so far are quite scanty and concern only the HCV genotype 3a. In fact, although most viral genotypes seem to activate members of the suppressors of cytokine signalling (SOCS) family in order to inhibit insulin signalling, in the case of genotype 3a, some in vitro observations are consistent with a downregulation of the PPAR*γ*. If confirmed, these observations may be relevant to the treatment of chronic hepatitis C, since insulin resistance is a factor of poor response to antivirals. This article will briefly review the role of PPARs in insulin resistance, the interactions between HCV and PPARs, and their clinical significance.

## 2. PPARs in Insulin Resistance and Diabetes

PPARs are the established targets of several classes of drugs used in the management of the metabolic syndrome, like the fibrates and the thiazolidindiones. A new class of pan-PPAR agonists, the glitazars, is presently under investigation. This article will describe the role and significance of PPAR*α* and *γ* in glucose metabolism, since the few data from HCV models have addressed the deregulation of only these two factors. No data are currently available about the involvement of PPAR*δ* in the HCV-associated insulin resistance.

PPAR*α* is principally expressed in tissues exhibiting high rates of *β*-oxidation such as liver, kidney, heart, and muscle, and can be activated by dietary fatty acids and eicosanoids or by specific drugs such as fibrates. Activation of PPAR*α* results in increased fatty acid *β*-oxidation in the liver [1], increased expression of HDL apolipoproteins Apo A-I and Apo A-II [2], inhibited expression of Apo C-III [3], increased lipoprotein lipase activity [4], and, therefore, increased VLDL and remnants clearance. As to insulin resistance, PPAR*α* seems to improve—indirectly—insulin sensitivity by increasing hepatic catabolism of lipids, and thus reducing lipid supply to skeletal muscle [5].

PPAR*γ* is expressed at very high levels in adipose tissue, and much less in the liver and other organs. Apart from its natural ligands, that is, fatty acids, it is activated by drugs of the class of thiazolidindiones such as rosiglitazone and pioglitazone. Hyperglycaemia and hyperlipidaemia in obese and diabetic animals are improved by pioglitazone through reduction of both peripheral and hepatic insulin resistance [6]. In the animal model, treatment with PPAR*γ* agonists lowers plasma level of free fatty acids and insulin, and increase the phosphorylation level of Akt at both threonine 308 and serine 473 in the liver and both the adipose and muscle tissues [7]. This, in turn, is correlated with tyrosine phosphorylation of insulin receptor *β* subunit and insulin receptor substrate-1, and serine phosphorylation of glycogen synthase kinase-3*α*/*β* [7]. Additional hepatic effects include increased insulin sensitivity via G-protein subunits downregulation, leading to reduced glucose production by approximately 30%, accompanied by a significant increase of glucose-induced insulin suppression in *β*-cells [8].

In addition to the direct effects on factors involved in lipid and glucose homeostasis, PPARs may have insulin sensitizing effects via their anti-inflammatory activity. PPAR*γ* reduces the expression of activator protein-1 and nuclear factor-*κ*B, reduces specific subsets of lipopolysaccharide and interferon (IFN) target genes in macrophages [9, 10], and reduces tissue expression of tumor necrosis factor-*α* (TNF*α*), IFN-*γ*, C-reactive protein, and induction of other proinflammatory cytokines, including hepatocytes [11].

Thus, treatment with PPARs agonists results in improved insulin sensitivity via diverse mechanisms, both direct and indirect, and both at the level of the liver and at the level of extrahepatic tissues.

## 3. Hepatitis C Virus Infection and Insulin Resistance

Insulin resistance and type 2 diabetes are common complications of all liver diseases, especially at the advanced stage. In the case of HCV infection, however, both clinical and experimental observations suggest that HCV may directly interfere with glucose homeostasis. Cross-sectional, case-control, and longitudinal studies—performed in both large unselected cohorts and in patients with liver or kidney transplantation—have suggested that type 2 diabetes may be more prevalent in chronic hepatitis C patients, especially if aged more than 40 and if other major risk factors of glucose intolerance are present [12]. The confounding effect of liver disease stage can be eliminated if studies are conducted in patients with little or no liver fibrosis, and if—instead of looking for cases with overt diabetes—glucose homeostasis is assessed as level of insulin sensitivity. Hui et al. [13] found that 121 HCV-infected patients with stage 0 or 1 liver fibrosis had higher levels of HOMA score compared with 137 healthy volunteers matched by sex, body mass index, and waist-to-hip ratio. This work proved that HCV may induce insulin resistance at early stages of liver diseases, and provided, in addition, the first evidence that this effect may be due to genotype-specific sequences.

Some recent work suggests a trend between HCV replicative level and level of insulin resistance [14]. The low-level correlation may be due to the fact that the global level of insulin resistance is likely to depend on the contribution from the adipose tissue and the muscle, two extrahepatic compartments that are not infected by HCV.

The effect of antiviral therapy is another classical way to prove an association between infection and disease. Romero-Gómez et al. have shown that both the level of insulin resistance [15] and the incidence of ex novo glucose intolerance over time [16] are reduced after successful therapy in chronic hepatitis C patients, whereas no improvement is observed in nonresponders. However, independent confirmation of these observations is warranted.

The association between HCV and insulin resistance has noteworthy consequences, clinically and conceptually. From the clinical standpoint, insulin resistance accelerates fibrogenesis [13, 17–19] and impairs response to IFN-*α*-based antiviral therapy [15, 20, 21]. In addition, HCV infection is an interesting example of insulin resistance not necessarily associated with other components of the metabolic syndrome, thus providing the framework for longitudinal studies on specific risk assessment. Last, but not least, the question arises what may be the advantage for HCV to increase insulin resistance; it is apparent that all HCV genotypes studied so far induce insulin resistance, albeit to a different extent [13, 14], suggesting some evolutionary constraints aimed at maintaining the insulin-resistant phenotype despite the viral genome sequence divergence over time.

Since HCV appears to directly interfere with the glucose homeostasis, several studies have tried to analyze in detail the potential interactions between viral products and insulin signalling. Experimental data suggest a direct interference of HCV with the insulin signalling cascade via proteasome degradation of the insulin receptor substrates -1 and -2 [22, 23]. HCV may also impair insulin signalling transduction indirectly, that is, through increased levels of proinflammatory cytokines such as TNF-*α* [24, 25]. The interference with the insulin signalling seems to proceed via HCV genotype specific mechanisms (see below) and insulin resistance levels vary according to the infecting HCV genotype, although all genotypes induce insulin resistance. Interestingly, intracellular factors dysregulated by HCV and responsible for the insulin resistant phenotype may play promiscuous effects as they are also involved in regulating IFN-*α* signalling. These factors include some members of the suppressor of cytokine signalling (SOCS) family [22, 23, 26] and the protein phosphatase 2A [27]. Thus, modulating the levels and/or the activity of these factors may not only reverse hepatic insulin resistance but also help establishing the IFN-*α*-induced antiviral state at the site of HCV replication. This is one of the reasons for trying to restore insulin sensitivity in chronic hepatitis C patients failing to respond to therapy (see below).

## 4. HCV Infection and PPARs Expression

**Figure 1:**
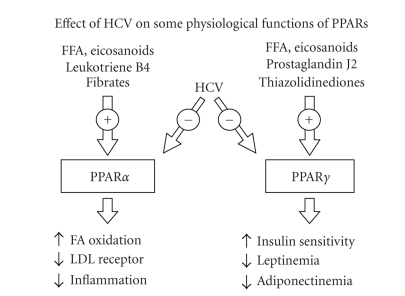
Reported effects of HCV on some of the relevant physiological functions of PPAR*α* and PPAR*γ*. The inhibitory effect on PPAR*γ* has been described only for the genotype 3a. For further explanations, please refer to the text.

Very little data is available concerning the interaction between HCV products and PPARs expression (Figure 1). PPAR*γ* mRNA level was measured in Huh-7 hepatoma cells transfected with the HCV genotype 3 core encoding sequence, and was found significantly decreased compared to untransfected cells. No changes were observed in cells transfected with the core protein 1b [28]. Incidentally, PPAR*α* was undetectable, even in untransfected cells, precluding further evaluations of an interaction with HCV, if any.

Cells transfected with the genotype 3a had both increased content of triglycerides [29] and reduced levels of IRS-1, leading to insulin resistance, as measured by reduced Akt phosphorylation following incubation with insulin [23]. The role of PPAR*γ* in HCV 3a-associated insulin resistance in vitro was further assessed by treating transfected cells with a PPAR*γ* agonist, rosiglitazone. Both IRS-1 protein and insulin-stimulated Akt phosphorylation levels increased after treatment with rosiglitazone of cells transfected with the core protein 3a. The recovery of the IRS-1 protein expression and Akt phosphorylation levels was, however, rather modest, that is, about 20% and 26%, respectively [23]. Additional recovery of both IRS-1 level and Akt activation was obtained by inhibiting SOCS-7 upregulation induced by the same viral genotype in this model [23]. Although cells expressing the HCV core 1b had reduced IRS-1 content and insulin resistance, the effect seemed to be mediated by both SOCS- and PPAR*γ*-independent mechanisms. Thus, based on this isolated observation, it seems that PPAR*γ* downregulation may be responsible, at least in part, for the insulin resistance observed in vitro upon expression of HCV genotype 3a core protein expression.

By analyzing total RNA extracted from the liver of chronic hepatitis C patients, de Gottardi et al. found that the transcription level of both PPAR*α* and PPAR*γ* was decreased in genotype 3 infection as compared to genotype 1 [28]. When patients were stratified according to the presence of steatosis, PPAR*α* and *γ* mRNA were reduced only in steatotic livers from patients infected with genotype 1. In those with genotype 3, both PPAR mRNA levels were always low, independently of the presence of steatosis. In this study, the two groups of patients with genotype 3 and 1 were comparable as to gender distribution, age, BMI, liver disease activity, and fibrosis stage. However, in this study, a direct correlation between PPARs levels and IR was not evaluated.

The level of PPAR*α* mRNA has been measured in the liver of chronic hepatitis C patients also in another study [30], and found to be profoundly suppressed (about 85% compared to control livers). The inhibition of PPAR*α* was paralleled by a significant decrease of the carnitine palmitoyl acyl-CoA transferase 1 mRNA, a key enzyme in the mitochondrial *β*-oxidation of fatty acids, and confirmed in an in vitro model expressing the HCV core protein [30]. These authors focused their discussion on the role of PPAR*α* as anti-inflammatory mediator, and on the consequences of its suppression in the pathogenesis of hepatitis C. It is important to remember that the impaired transcriptional activity of PPAR*α* associated with HCV infection may indirectly worsen IR via increased expression of TNF-*α*; thus, the role of inflammation in the pathogenesis of HCV-associated IR should not be overlooked [31]. On the contrary, it is unlikely that HCV-induced steatosis, due to decreased expression of PPAR*α* and several other mechanisms [32], may aggravate insulin resistance, since the latter has been convincingly shown to precede steatosis, and not vice versa [33].

## 5. Perspectives for Treatment

As said above, insulin resistance reduces the rate of response to antivirals in chronic hepatitis C [15, 20, 21]. A sustained virological response (SVR) was observed in 23 of 70 (32.8%) of patients with genotype 1 and insulin resistance (i.e., with a HOMA > 2) versus 26 of 43 (60.5%) of genotype 1 patients without insulin resistance (*P* = .007) [15]. These findings were independently confirmed [20] and extended to nonresponders with genotypes 2 and 3 [21]. Thus, it was suggested that insulin resistance should be corrected in patients with chronic hepatitis C not responding to IFN-*α*-based treatment, in order to improve response upon retreatment. The modalities of this intervention, however, have not been established. In addition, the optimal HOMA-IR score to be reached has not been identified.

A recent prospective, multicenter study aimed at investigating the efficacy and safety of the insulin-sensitizer pioglitazone, 15 mg QD, added to the pegylated IFN-*α*_2a_, 180 *μ*g QW/ribavirin, 1 000–1 200 mg QD combination therapy in chronic hepatitis C patients who were previously nonresponders to a pegylated IFN-*α*/ribavirin combination without the insulin sensitizer [34]. All patients had a baseline HOMA > 2 as additional inclusion criterion, because this was the threshold discriminating responders from nonresponders in previous works [16, 21]. Diabetic patients were however excluded. Unfortunately, none of the first five patients enrolled into the trial had a satisfactory virological response after 12 weeks of retreatment, despite the fact that in at least three of them the insulin resistance score improved, and thus the study was prematurely terminated [34].

Additional data on this topic have been presented at the 2008 annual meeting of the American Association for the Study of Liver Diseases. In an interim analysis of a clinical trial, 30 mg QD of pioglitazone were given for four weeks as monotherapy and then added for the first four weeks of a standard therapy of treatment-naïve, nondiabetic chronic hepatitis C patients. The authors showed that the triple regimen containing pioglitazone increased significantly the rate of virological response after 4 weeks of therapy compared to pegylated IFN-*α*/ribavirin combination [35]. Long-term data are eagerly awaited. However, in another randomized, double-blind, placebo-controlled study, adding pioglitazone 30 mg QD simultaneously to the standard of care (i.e., without a preceding administration as monotherapy) clearly increased the on-treatment virological response, but failed to increase the sustained virological response after the end of treatment [36]. Additional studies evaluating different schedules are clearly warranted. This approach, however, should also take into consideration the known effects of PPAR agonists on serum lipid profile and their potential consequences on the HCV life cycle. HCV circulates bound to lipoproteins in complexes known as lipoviroparticles [37]. As a result, HCV entry into hepatocytes appears to be mediated and facilitated, among others, by the LDL receptor [38]. In keeping with this, at least two recent studies have suggested that baseline LDL-associated cholesterol levels may affect response to antiviral therapy [39, 40]. In fact, higher levels of cholesterol- and ApoB-rich lipoproteins could facilitate viral clearance by impeding HCV interaction with cell surface receptors. Thus, drugs like thiazolidinediones that modify the circulating lipoprotein profile may have unexpected—and potentially unwanted—effects on the HCV life cycle. On the other hand, in addition to lipid-lowering effects, PPAR*α* agonists have been shown to decrease also the expression of the LDL receptor in experimental models [41]. This may offset the untoward effect on lipoproteins by impairing HCV entry into target cells. Although highly speculative, these hypotheses deserve being appropriately evaluated in clinical trials.

Modulating the levels and/or the activity of intracellular factors involved in HCV-induced insulin resistance may not only reverse hepatic insulin resistance but also help establishing the IFN-*α*-induced antiviral state at the very site of HCV replication.

However, specific inhibitors of SOCS family members and of the protein phosphatase 2A are either not suitable for in vivo administration or toxic. Alternatively, increasing insulin sensitivity may be achieved by modulating serum levels of specific cytokines, such as TNF-*α*, associated with insulin resistance [24, 25], but the administration of anti-TNF-*α* antibodies to chronic hepatitis C patients may be risky [42]. Insulin sensitizers may also inhibit HCV replication by decreasing serum free fatty acids flow to hepatocytes; saturated and monounsaturated free fatty acids have indeed been shown to stimulate HCV replication in an in vitro model [43].

Thus, although increasing insulin sensitivity may be a rational option in chronic hepatitis C patients not responding to current combination therapy, more work is warranted to identify the appropriate treatment schedule. It is not clear whether the best approach would be using a thiazolidindiones, activating PPARs, or a biguanide such as metformin, whose mechanism of action is specifically directed on the hepatic AMP-activated protein kinase [44]. Another major issue concerns the treatment schedule. It is unclear whether one should start the antiviral retreatment together with the insulin sensitizer or only once the HOMA-IR score has decreased to a level predicting a sufficient SVR rate [16]. As an alternative, higher dosages of thiazolidindiones (as compared to the above quoted study) or metformin may be used. Finally, insulin sensitizing therapy might need to be tailored according to HCV genotype, and PPARs agonists should probably be considered only in insulin-resistant patients with HCV genotype 3a [23]. Finally, interactions of insulin sensitizing agents with other drugs, most notably those taken for psychiatric comorbidities, should be considered. In the quoted study, a paradoxical increase of the HOMA score was observed in two patients during therapy, and both patients were taking drugs for their psychiatric comorbidities that may also alter insulin sensitivity, that is, zopiclone and olanzapine. Thus, the beneficial effect of pioglitazone may be nullified in patients taking drugs for psychiatric indications. In addition to the potential effects of these and other drugs, also the acute administration of IFN-*α* may induce some degree of insulin resistance in both healthy subjects [45] and chronic hepatitis C patients [46]. The interactions among all these drugs are, however, speculative, but require further studies, in view of the frequent use of antidepressants in chronic hepatitis C patients. Thus, further clinical trials aiming at reducing the insulin resistance in chronic hepatitis C via different pharmacological interventions are warranted.
